# Control of Multiferroic properties in BiFeO_3_ nanoparticles

**DOI:** 10.1038/s41598-019-39517-3

**Published:** 2019-02-28

**Authors:** Diego Carranza-Celis, Alexander Cardona-Rodríguez, Jackeline Narváez, Oscar Moscoso-Londono, Diego Muraca, Marcelo Knobel, Nancy Ornelas-Soto, Andreas Reiber, Juan Gabriel Ramírez

**Affiliations:** 1 Department of Physics, Universidad de los Andes, Bogotá, 111711 Colombia; 2 Instituto de Física ‘Gleb Wataghin’, Universidade Estadual de Campinas (UNICAMP), CEP13083-859 Campinas, São Paulo Brazil; 3 Facultad de Ingeniería, Universidad Autónoma de Manizales, Manizales, Colombia; 4 Tecnologico de Monterrey, Escuela de Ingeniería y Ciencias, Ave. Eugenio Garza Sada 2501, 64849, Monterrey, N.L., Mexico; 5 Department of Chemistry, Universidad de los Andes, Bogotá, 111711 Colombia

## Abstract

BiFeO_3_ (BFO) nanoparticles (NPs) were synthesized using the sol-gel method at different calcination temperatures from 400 °C to 600 °C. XRD studies have confirmed that all BFO NPs show distorted rhombohedral crystals that match the R3c space group. We found evidence of local structural strain that develops with increasing particle size as suggested by TEM and Raman spectroscopy measurements. Magnetic measurements suggest that NPs have two distinct regimes: a ferromagnetic-like one at low temperatures and a superparamagnetic-like one at room temperature. The crossover temperature increases with NPs size, suggesting a size-dependent blocking magnetic regime. Similarly, local piezoelectric measurements at room temperature in single NP have confirmed a ferroelectric order with a NP size-dependent d_33_ coefficient. An analysis of both the ferroelectric and the magnetic results suggest that ferromagnetism and ferroelectricity coexist at room temperature in NPs. Our results lead to the possibility of tailoring the ferroic order in multifunctional materials by means of NP size.

## Introduction

Multiferroic materials have attracted great interest because they allow, in principle, the manipulation of magnetic order with electric fields and electrical polarization with external magnetic fields^1^. This characteristic is known as magnetoelectric (ME) coupling and it provides a new degree of freedom in the next generation of low-dimensional devices (memories, sensors, solar cells, etc.)^2–4^. ME coupling has been shown to be strong in composite materials, but a definite understanding in single-phase materials is still lacking. In most single-phase materials the ME coupling is either quite weak or it does not occur. Hence, the fabrication of single-phase magnetolectric materials with a strong coupling at room temperature is a current challenge and a rather active research topic.

There are only few single-phase magnetoelectric materials at room temperature. One of them is the bismuth ferrite, BiFeO_3_ (BFO)^5^. BFO is an archetypical ferroelectric multifunctional oxide with rhombohedral distorted perovskite structure that belongs to the R3c space group. BFO displays a Dzyaloshinskii-Moriya (DM) interaction among nearest neighbor Fe^3+^ spins^6^ that produces an antiferromagnetic long-cycloid spin structure of wavelength 62 nm^7^. In addition, DM interaction is the responsible for the ME coupling^8^. BFO has a high Néel temperature (640K) and high Curie transition temperature (1100K), as well as high polarization of Ps = ~100 μC/cm^2^ in the [111] crystallographic direction. Bulk BFO is also known by its weak ME coupling^9^. However, low-dimensional confinement is expected to lead to (*i*) a strengthening of the ME coupling^10^ and (*ii*) a ferromagnetic-like behavior^11^. The latter is caused by particle sizes smaller than their long-cycloid spin structure^6,7^.

In this paper, we report the synthesis of BFO NPs by sol-gel method at different calcination temperatures. Particles have a rhombohedral crystal structure as determined by X-Ray diffraction and Raman spectroscopy. The NPs morphology was investigated by transmission and field emission electron microscopy (TEM and FE-SEM). Low-temperature magnetic characterization revealed that the NPs are ferromagnetic. By increasing the calcination temperature, the sample magnetization decrease, regardless of the magnetic moment per particle increases, as calculated by fitting of the isothermal magnetization hysteresis loops to a magnetic particle model. Remarkably, our local measurements of the ferroelectric behavior on individual NPs by piezoelectric force microscopy (PFM) shows directly that the piezoelectric properties scale with size. Hence, we have found that BFO NPs are multiferroic in which a ferromagnetic order was induced. These results demonstrate that the NP size could be used as a tuning parameter to control the multiferroic order in BFO NPs.

## Methods

BFO NPs were prepared by sol-gel method^12^; using an appropriate amount (to obtain a molar concentration of 0.0025 M in 25 mL of solution) of bismuth nitrate pentahydrate (Bi(NO_3_)_3_·5H_2_O) which was dissolved in 8 mL of deionized water and 2 mL of glacial acetic acid (CH_3_COOh); it was stirred at room temperature for 24 h. Then, deionized water was added until achieving a total of 25 mL of solution and the required amount of iron nitrate nonahydrate (Fe (No_3_)_3_·9H_2O_) to obtain 0.0025 M of the molar concentration of this reagent. This solution was stirred for 3 h and afterwards 0.5 mmol of citric acid (C_6_H_8_O_7_) was added while the temperature was increased to a range between 70 °C and 75 °C. Then, the pH of the solution was controlled by immediately adding drops of ammonium hydroxide until the solution turned into a translucid red.

Afterwards, the solution was continuously stirred while keeping the temperature fixed for another 8 hours. After that, 1.25 mmol of etylenglicol (C_2_H_6_O_2_) were added at 90 °C, which produce the precursor gel. This gel was calcined for 3 h in an oven at different calcination temperatures, from 400 °C to 630 °C. Finally, the resulting powder was washed out several times with deionized water and glacial acetic acid and let to dry at room temperature.

The crystal structure of the powder was determined by X-Ray diffraction (XRD). We used Cu-k_α_ radiation and angular range of 2θ from 5 to 120 with step size 0.002 degrees in order to perform a meaningful Rietveld refinement using the Profex software^13^. Raman spectroscopy was measured in the range of 50 cm^−1^ to 1000 cm^−1^ to characterize typical resonant modes found in BFO. The local structure and particle size were characterized by transmission electron microscopy (TEM) using a JEOL TEM-FEG (JEM 2100F, 300KV) and JEOL TEM-MSC (JEM 2100, 200KV). The images were acquired using a Gatan/Orius SC600/831 camera at different magnifications and analyzed using Gatan Micrograph software. The samples were prepared before the experiment by drying a drop of a dispersion on ultrathin carbon film supported on holey carbon (Ted Pella). The particle size also was characterized by field emission scanning electron microscopy (FE-SEM) Tescan LYRA 3. To image the particles, we dissolve them into isopropyl alcohol on an Al foil. The magnetization hysteresis curves (*M* vs *H*) at room temperature were obtained with a Lakeshore™ vibrating sample magnetometer (VSM) and a Quantum Design™ SQUID magnetometer. Topography of individual NP was obtained with an Asylum Research MFP-3D™ AFM. The ferroelectric characteristics were measured in piezoelectric force microscopy (PFM) mode and switching spectroscopy (SS-PFM) mode^14^. The ferroelectric hysteresis loops on single BFO NP were made with a probe of Asylum AC240TM-R3 with PtIr tip coating. BFO NPs were dissolved into isopropyl alcohol and dispersed using the spin coating onto an Au/Ti/SiO_2_/Si and Ag/Ti/SiO_2_/Si substrates to measure simultaneously the topography and ferroelectric characteristics of individual particles.

## Results and Discussion

**Figure 1:**
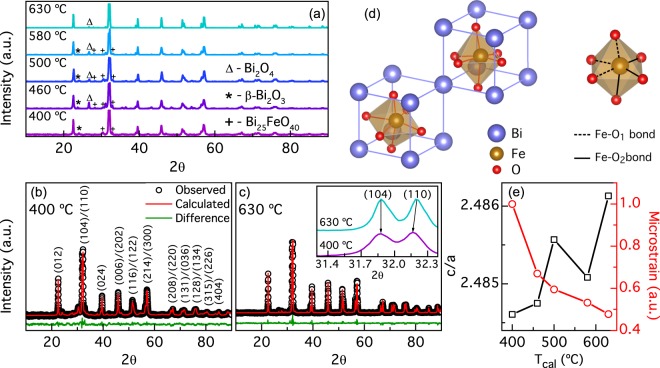
(**a**) X-ray diffractograms of BFO NPs calcined at different temperatures. Dominant secondary phases Bi_2_O_4_, β-Bi_2_O_3_ and Bi_25_FeO_40_ are indicated with Δ, * and + symbols respectively. Less intense secondary phases *ε-*Bi_2_O_3_ and Bi_2_Fe_4_O_9_ are not indicated but they were also identified and included in diffractograms Rietveld refinements. Rietveld refinements for 400 °C and 630 °C are shown in (**b**) y (**c**). *Inset*: Diffractogram zoom around 2*θ* ~ 32° for samples calcined at 400 °C and 630 °C showing the reflections of (104) and (110) planes. (110) corresponding peak is shifted to lower angles as NP size decreases, as the arrow indicates. Typical R3c rhombohedral reflections planes for BFO are indicated in (**b**,**d**) BFO perovskite unit cell in pseudocubic representation. Octahedral organization of Fe and O ions is showed indicating Fe-O bonds. (**e**) Lattice parameters (calculated with refinements) ratio *c/a* and microstrain (roughly estimated without the instrumental broadening) as functions of *T*_*cal*_ indicating increment of strain with NPs size decrease.

Figure 1(a) shows the XRD pattern for BFO NPs calcined at five different temperatures (*T*_*cal*_) from 400 °C to 630 °C. In all samples, the peaks coincide with planes expected for BFO. Additional peaks can be attributed to secondary phases formed in the fabrication process. Rietveld refinements of the XRD patterns for nano-powders calcined from 400 °C to 630 °C are shown in Fig. 1(b,c) and Supplementary Fig. S1. Detailed analysis of the refinement is shown in Supplementary Fig. S2 and in Supplementary Table S1. In Fig. 1(b) each peak is labeled with its corresponding Miller indices in hexagonal representation^15^. The typical double peaks (104)/(110) around $2\theta =32^\circ$ from the BFO NPs calcined at 400 °C and 630 °C are shown in the inset of Fig. 1(c). The (110) peak shifts toward lower angles in the sample calcined at a temperature of 400 °C, attributed to an increment of the crystal strain caused by NPs size confinement^16^. The BFO phase percentage obtained from the refinement was 70.7% and 90.5% for 400 °C and 630 °C, respectively (see Supplementary Fig. S2). The refinements confirm that our NPs have a rhombohedral perovskite structure with an R3c space group symmetry (Chi-square fittings are: ${\chi }_{630}^{2}=2.4$ and ${\chi }_{400}^{2}=2.9$). Figure 1(d) shows a schematic construction of a BFO unit cell in a pseudocubic system. The perovskite structure, characterized by the octahedral coordination, can be observed. In the NPs calcined at 400 °C, the lattices parameters calculated were a = b = 5.5850 Å, and c = 13.8764 Å; while for the NPs calcined at 630 °C were a = b = 5.5810 Å, and c = 13.8751 Å, which are in close agreement with bulk BFO reports^15^. Figure 1(e) shows the evolution of the ratio *c*/*a* and microstrain vs. *T*_*cal*_. The microstrain was roughly estimated using the Williamson-Hall equation^17,18^ without including the correction due to instrumental broadening and normalized to the highest value, with the purpose of seeing the strain evolution trend across the samples. This anticorrelation between *c/a* ratio and microstrain shows that our NPs exhibits variations in the degree of crystal strain that decreases with *T*_*cal*_.

**Figure 2:**
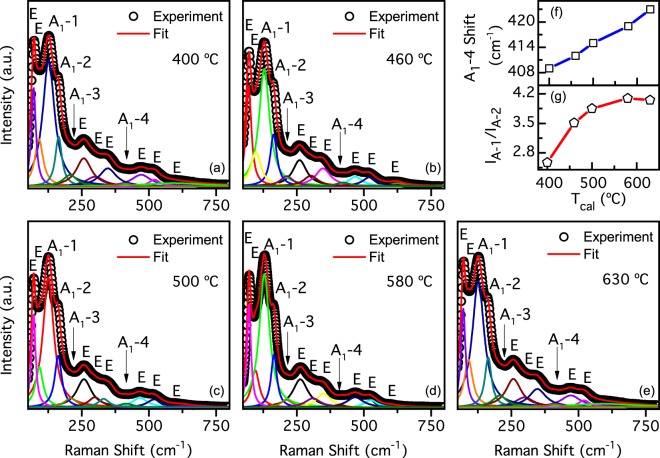
Raman spectra of NPs calcined at (**a**) 400 °C, (**b**) 460 °C, (**c**) 500 °C, (**d**) 580 °C and (**e**) 630 °C. Symbols represent observed spectra in each figure. Figures also show spectra fitting curves and deconvoluted individual peaks that have been labeled with corresponding indices according to simetry of raman modes expected for the BFO. (**f**) Red shift of A_1_ − 4 peak with *T*_*cal*_ increase. (**g**) A_1_ − 1 peak integral intensity increase with higher calcination temperature.

Figure 2 shows the Raman spectra obtained at room temperature for the studied samples. We have identified 12 active modes (4 A_1_ and 8 E) out of 13 modes present in bulk (4A_1_ + 9E) in the Raman spectra by a peak-fitting procedure using Lorentzian distributions. Raman spectra of our NPs, for each *T*_*cal*_, are in good agreement with those expected for rhombohedral BFO with a R3c space group. Therefore, we confirm that our NPs have the ferroelectric R3c phase. Because there are not considerable changes between the Raman spectra, we could infer that ferroelectric phase is present in all samples^19,20^. However, an unexpected red shift of all A_1_ peaks from the typical values reported for bulk BFO can be observed. Furthermore, such red shift increases with decreasing *T*_*cal*_ (see Supplementary Table S2) and with respect to BFO bulk single crystal reported Raman shifts^20^. For example, in the sample calcined at 400 °C, we observe a red shift of 15.6% for the A_1_ − 1 Raman mode, 8.5% for A_1_ − 2 while for A_1_ − 3 and A_1_ − 4 we observe red shifts of 7.9% and 16.5%, respectively. In Fig. 2(f) we show the A_1_ − 4 mode red shift evolution as a function of *T*_*cal*_; such mode is associated to Fe-O1 and Fe-O2 bonds^21,22^ (see Fig. 1(f)). From these results we can identify a possible distortion of Fe-O bonding distances or Fe-O-Fe bonding angle and relate them with the calcination. Indeed, such red shifts observed in Raman spectra and the analysis of the Williamson-Hall equation from XRD results could be related to the strain of NPs and the size confinement. Similarly, A_1_ − 1 Raman peak suppression can be associated to an increase of the magnetoelectric coupling in BFO^10^. In our samples, such suppression increases with decreasing *T*_*cal*_, as shown in Fig. 2(g) where the A_1_ − 1 integral intensity *I*_*A*1_ (normalized to the A_1_ − 2 integral intensity *I*_*A*2_) increase with *T*_*cal*_.

**Figure 3:**
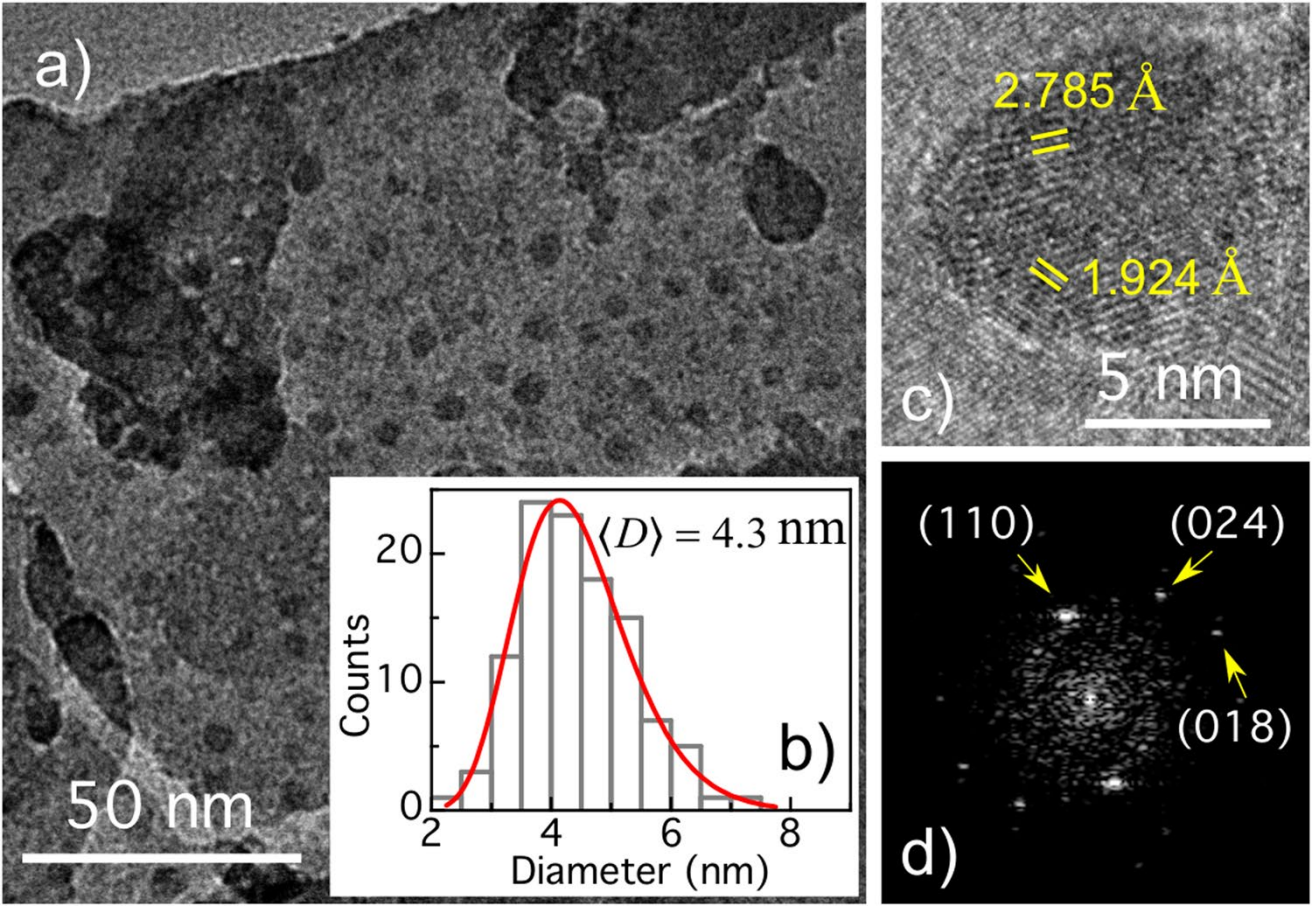
(**a**) TEM image of BFO NPs calcined at 400 °C. *Inset:* Histogram of the NPs size distribution obtained from the TEM image. The lognormal fit is also shown. (**c**) TEM image of a single nanoparticle and (**d**) corresponding Fourier transform.

Figure 3(a) shows a TEM image of NPs calcined at 400 °C. Using this image, as well as others from several TEM images, the size distribution presented in Fig. 3(b) was built. The diameter distribution was fitted with a lognormal-type function, from which a mean diameter close to 4.3 nm was determined, as shown in the figure. High resolution TEM image of NP with nearly 10 nm diameter is shown in Fig. 3(c). Interplanar Bragg distances 2.785 Å and 1.924 Å can be identified in the NP, which are in good agreement with the distance between (110) and (024) lattice planes of BFO. From FFT of TEM image (see Fig. 3(d)), a third spot appears, whose interplanar distance of 1.632 Å coincide with that of (018) BFO lattices planes, additionally to (110) and (024) planes observed on high resolution image^15^. Furthermore, Supplementary Fig. S3 shows the size distribution, for the NPs calcined at 630 °C, extracted from several FE-SEM images like that showed in the inset. From the lognormal-type function fitting we obtain a mean particle diameter close to 54 nm.

**Figure 4:**
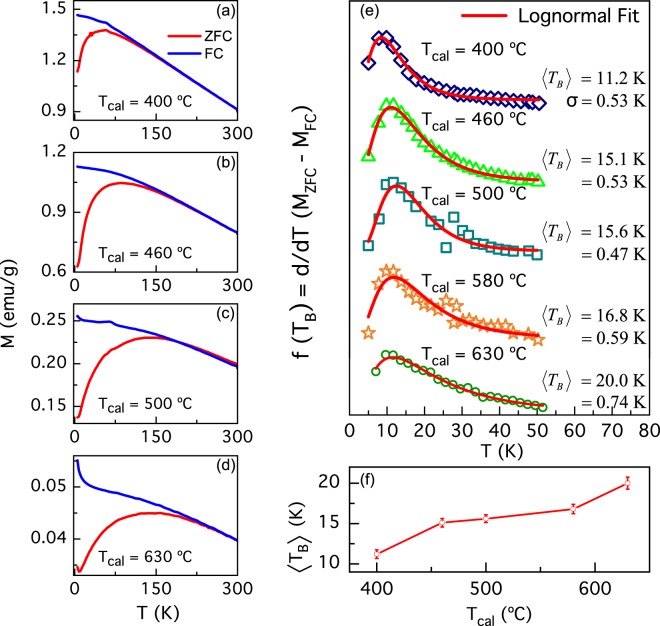
(**a**–**d**) ZFC-FC magnetization curves of the NPs for *H* = 1 kOe. (**e**) Blocking temperature obtained from the derivative method and lognormal fittings for (**a**–**d**) ZFC-FC curves. (**f**) Mean blocking temperature *T*_*B*_ as a function of the calcination temperature of the NPs.

Figure 4 shows the zero-field cooling and field cooling (ZFC-FC) magnetization curves for *H* = 1 kOe applied field. For all samples, the FC magnetization decreases monotonically, while the ZFC curves reach a maximum (*T*_*max*_), which appears at higher temperatures with increasing *T*_*cal*_. The ZFC curves resemble the ones of magnetic nanoparticles with a superparamagnetic behavior. Thus, the temperature at which the maximum of the ZFC magnetization occurs can be ascribed to a blocking temperature (*T*_*B*_), *i*.*e*., this temperature is closer to the one in which the system undergoes a blocking-to-unblocking transition. Upon increasing temperature, the ZFC and FC curves merge few degrees above the maximum reached by the ZFC curve. This point, known as the irreversibility temperature (*T*_*i*_), can be interpreted as an indicative of the existence of a blocking temperature distribution *f*(*T*_B_), associated to a NP size distribution^23,24^. We estimated *f*(*T*_*B*_) in each sample using the reduced-magnetization derivative, calculated as $\frac{d}{dT}({M}_{ZFC}-{M}_{FC})\,$^25^. Figure 4(i) shows *f*(*T*_*B*_) distributions for all samples and it can be noticed that they can be well fitted with a lognormal-type function. The $\langle {T}_{B}\rangle$ obtained range from 11.2 K for the sample calcined at 400 °C to 20.0 K in sample calcined at 630 °C. The dependence of $\langle {T}_{B}\rangle$ with *T*_*cal*_ is plotted in Fig. 4(j). An increase of the Néel relaxation time could explain the increment of $\langle {T}_{B}\rangle$ with *T*_*cal*_ in our NPs. Thus, the blocked-superparamagnetic transition temperature increases with NP size^26^, and therefore suggests that the NPs have a nearly superparamagnetic behavior.

**Figure 5:**
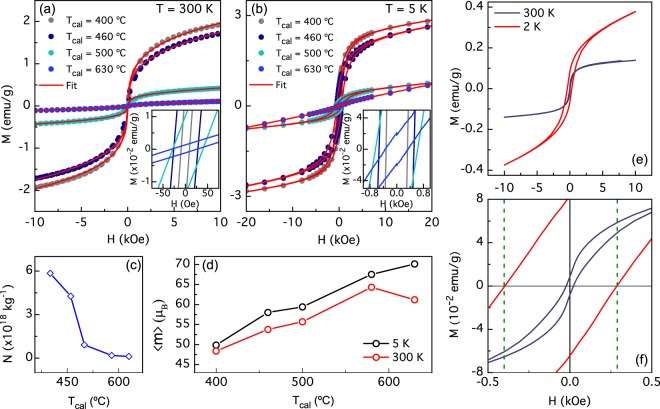
(**a**) Magnetic hysteresis loops taken at room temperature and (**b**) at 5 K of BFO NPs prepared at different calcination temperatures (*T*_*cal*_). The red line represents the fit using a Langevin equation. (**c**) Best fitting-parameters for *N* (number of NPs per mass unit) and (**d**) *m* (particle magnetic moment in Bohr magnetons units) with the calcination temperature for *M* vs. *H* experimental curves taken at 300 K and 5 K. (**e**) M vs H hysteresis at 2 K and 300 K curve for a NPs sample calcined at 600 °C. (**f**) Hysteresis loop zoom; 2 K curve exhibits exchange bias effect.

Figure 5(a) shows the isothermal magnetization hysteresis loops (*M* vs. *H*) measured at 300 K for the studied BFO NPs. We found a *S*-shape and almost null values of the coercive field (*H*_C_) resembling a near superparamagnetic-like behavior, which confirm that observed at ZFC-FC curves. At higher fields, up to 3 Tesla (not shown), the magnetization does not to reach saturation indicating the existence of a paramagnetic-like component, which is possibly related to those disordered magnetic moments located at the NP surface. The NPs magnetization increases with decreasing *T*_*cal*_. In contrast, coercive field (*H*_C_) drastically increase at low temperatures, as shown in Fig. 5(b) for *T* = 5 K and can be attributed to a higher contribution from a ferromagnetic order. It can be noticed also that the paramagnetic component is present in all samples.

We performed fittings of the *M* vs. *H* curves using a modified Langevin function (to include a *H*_C_ dependence) where we take into account a lognormal-type distribution magnetic moments^27^ as suggested by our TEM and FE-SEM images and magnetic data. At last, we include a linear parameter with the field in the fitting calculations to take into account the paramagnetic contributions. Best fitting results are shown as continuous solid red lines in Fig. 5(a,b). Whit this procedure we were able to obtain the average magnetic moment per NP, $\langle m\rangle$, and number of NPs per unit mass, *N* (see Supplementary Table S3).

From Fig. 5(c) one can infer that *N* decreases monotonically with *T*_*cal*_. On other hand, Fig. 5(d) shows the evolution of $\langle m\rangle$ as function of ${T}_{cal}$, at 300 K and 5 K. In both cases, an increase of $\langle m\rangle$ with *T*_*cal*_ is observed. This means that $\langle m\rangle$ increase with NP size as opposite to the total magnetization. Such behavior can be understood assuming that at a given NP size, exceeding the long-cycloid spin structure, exhibits an antiferromagnetic core surrounded by a shell of uncompensated spins. Such magnetization-moment anticorrelation can be due to a surface to volume ratio effect^10^.

Additionally, Fig. 5(e) shows magnetization hysteresis loops at 300 K and 2 K, on BFO NPs calcined at 600 °C. Room temperature hysteresis loop exhibit superparamagnetic-like behavior with a very week ferromagnetic component (as stated before), with a 22 Oe coercive field. Regarding to low temperature hysteresis loop, coercive field increases to 340 Oe and NPs magnetism become predominantly ferromagnetic. Interestingly, a closer inspection of the magnetization, near zero field, shows a horizontal shift, see Fig. 5(f). Such a phenomenon is usually associated with an Exchange Bias (EB) effect, originated from the spins interaction at the interface between ferromagnetic and antiferromagnetic layers^28^. This effect is observed at low temperatures close 2 K. In our NPs, such effect could be associated with a core-shell structure of NPs with an antiferromagnetic core and a ferromagnetic shell. This effect have been observed in BFO NPs^29,30^. EB field has been calculated with the left (*H*_*C*1_) and right (*H*_*C*2_) coercive fields as ${H}_{EB}=-\,({H}_{c1}+{H}_{c2})/2$. The hysteresis loop taken at 2 K has a *H*_*EB*_ of 56 Oe, while *H*_*EB*_ taken at room temperature is zero.

**Figure 6:**
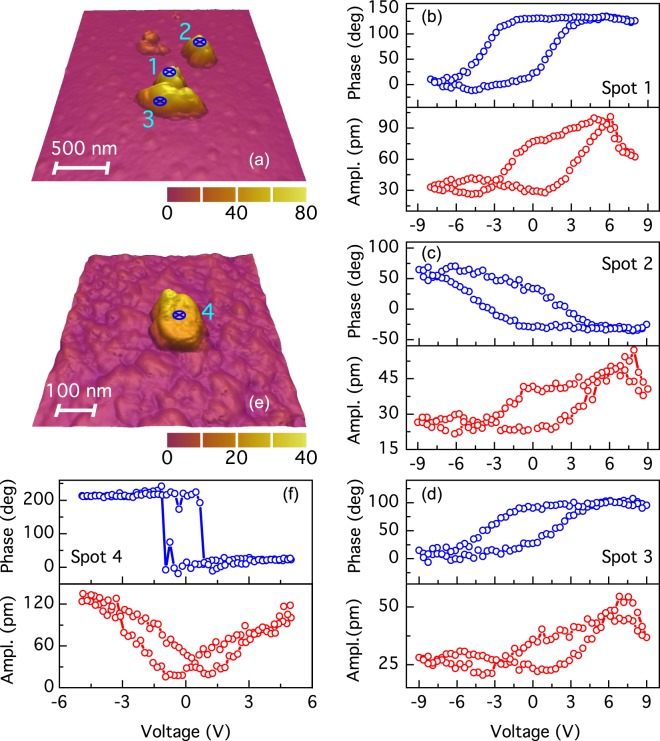
(**a**) AFM topography image of BFO NPs calcined at 630 °C. Spots on which hysteresis PFM curves were measured are shown. Curves (**b**–**d**) are the phase (blue) and amplitude (red) ferroelectric hysteresis loops corresponding to spots 1 to 3 on BFO nanoparticles, respectively. (**e**) AFM topography image of nearly 25 nm BFO NP calcined at 600 °C. On marked spot was measured (**f**) phase and (**g**) amplitude piezoelectric hysteresis loops.

Figure 6(a) shows AFM topography of dispersed BFO NPs calcined at 630 °C on an Au/Ti/SiO2/Si substrate. Such NPs agglomerations have lateral sizes of several hundred nanometers, while thickness ranges from 45 nm to 70 nm, approximately. We have performed local measurements of amplitude and phase piezoelectric hysteresis curves, on marked spots inside the AFM picture employing the switching-spectroscopy PFM method (SS-PFM)^14^, results are shown in Fig. 6(b,d). Phase hysteresis loops correspond to ferroelectric behavior with coercive fields between 2.55 V and 2.65 V. The small difference in values of the coercive fields can relate to the NP size or to the direction polar vector for switching. Interestingly the polarization phase switching is near to 140° and approximately to 109° and 71° in Fig. 6(d,e), respectively. Such values are consistent with the values predicted by the switching phase mechanism expected for rhombohedral BFO^7^. We calculate the area of the phase-voltage hysteresis loops^14^, for spots 1 to 3 and obtained the respective values 764.8 a.u., 548.9 a.u., and 499.4 a.u. corresponding to NPs of 70 nm, 50 nm and 45 nm sizes. It can be noted that the area mostly decreases with the NP size. The corresponding piezoelectric amplitude hysteresis for each spot, taken simultaneously, show typical butterfly shape loops corroborating the ferroelectric properties of BFO NPs. From these we calculated the local longitudinal piezoelectric coefficient d_33_ for spots 1, 2 and 3, the respective values are nearly to 7.74 pmV^−1^, 2.48 pmV^−1^ and 2.16 pmV^−1^. Thus, d_33_ coefficient mostly decrease with the NPs size similar to the switching area. The above results suggest that the ferroelectric switching can be tuned with the NP size. In addition, Fig. 6(e,f) shows the AFM topography and local PFM piezoelectric hysteresis loops of NPs calcined at 600 °C and dispersed on a Ag/Ti/SiO_2_/Si substrate. Piezoelectric curves were taken on the spot 4 marked on the topography image of the NP with size close to 25 nm. Phase hysteresis loop have a coercive field close to 1.5 V and a phase switching near to 180 °C corresponding to a ferroelectric behavior. Regarding to the amplitude piezoelectric hysteresis loop, this local measurement exhibits a typical and well-shaped butterfly. Calculating the coefficient d_33_ we obtain a value near to 13.6 pmV^−1^. This can be observed a change in the coercive fields as well as d_33_ coefficient regarding to the calcined at 630 °C sample results. The probable cause of such behavior is the change of bottom electrode from Au to Ag, changing with this the depletion layer at the electrode-NP interface and thus the measured piezoelectric properties^31^.

## Summary

In summary, we successfully synthesized BFO nanoparticles using the sol-gel method. Structural characterization suggests NPs have a distorted rhombohedral crystal lattice due to strain effects. The strain increases with decreasing calcination temperature, suggesting smaller particles have stronger strain. The NPs have been observed to be magnetically polarizable and that its magnetization increases with decreasing particle size. At low temperatures, the NPs show ferromagnetic order; at room temperature, they show a closer superparamagnetic-like behavior. Furthermore, the blocked-superparamagnetic transition, related to the observed blocking temperatures, suggests that this evolution take place at larger temperatures as NPs size increases. We have observed in the 600 °C sample, a slight shift of the hysteresis loops at 2 K, suggesting a small exchange bias effect. A possible scenario can be an antiferromagnetic-ferromagnetic coupling in a core-shell structure. Finally, we have corroborated that NPs are ferroelectric through piezoelectric hysteresis loops measurement on individual NPs and found that d_33_ coefficient increases with NP size as well as the total switching area. Our results allowed us to conclude that the BFO nanoparticles are multiferroic, *i*.*e*. ferroelectric and ferromagnetic, in contrast to its bulk behavior, where the magnetic coupling is known to be antiferromagnetic. These results open an avenue to control the multiferroicity order using strain resulting from the NPs confinement.

## Supplementary information

Supplementary information

## Data Availability

The data that support the findings of this study are available from the corresponding author upon request.
