# Supplementary material for: Control of Multiferroic properties in BiFeO3 nanoparticles
Source: Sci Rep. 2019 Feb 28;9:3182. doi: 10.1038/s41598-019-39517-3 (PMC6395681; doi:10.1038/s41598-019-39517-3)
Supplement: Supplementary file 1 — Supplementary information [file 41598_2019_39517_MOESM1_ESM.pdf]

Supplementary materials for "Control of Multiferroic properties in $BiFeO_3$ nanoparticles"

Diego Carranza-Celis[1], Alexander Cardona Rodríguez[1], Jackeline Narváez[1], Oscar Moscoso[2,3], Diego Muraca[2], Marcelo Knobel[2], Nancy Ornelas-Soto[4], Andreas Reiber[5], Juan Gabriel Ramírez[1],

[1]Department of Physics, Universidad de los Andes, Bogotá 111711, Colombia
[2]Instituto de Física 'Gleb Wataghin', Universidade Estadual de Campinas (UNICAMP), CEP13083-859 Campinas, São Paulo, Brazil
[3]Facultad de Ingeniería, Universidad Autónoma de Manizales, Manizales, Colombia
[4]Tecnologico de Monterrey, Escuela de Ingeniería y Ciencias, Ave. Eugenio Garza Sada 2501, 64849 Monterrey, N.L., México
[5]Department of Chemistry, Universidad de los Andes, Bogotá 111711, Colombia

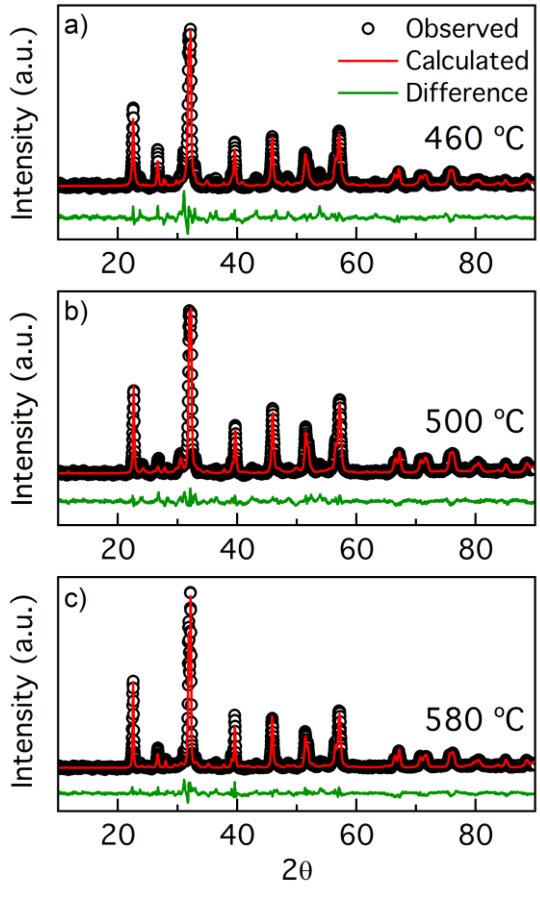

Figure S1. X-ray diffractograms and Rietveld refinement for NPs calcined at (a) 460 ºC, (b) 500 ºC and (d) 580 ºC

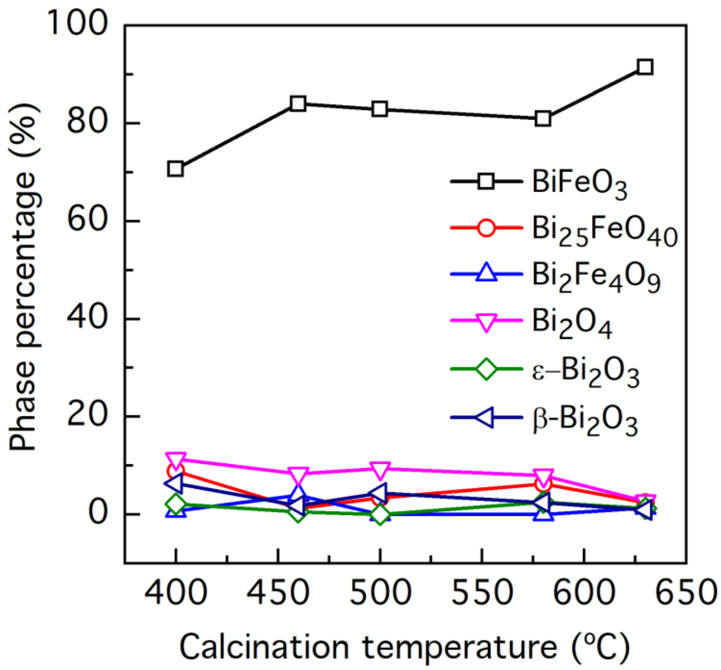

Figure S2. Phases employed in Rietveld refinement and its variation with the calcination temperature according to Rietveld refinements.

Table S1. Parameters obtained from Rietveld refinements for $BiFeO_3$ phase.

	400 ºC	460 ºC	500 ºC	580 ºC	630 ºC
χ^2	2.9	9.9	5.3	6.4	2.4
a, b (Å)	5.5585	5.5804	5.5834	5.5807	5.5810
c (Å)	13.8764	13.8659	13.8780	13.8684	13.8750

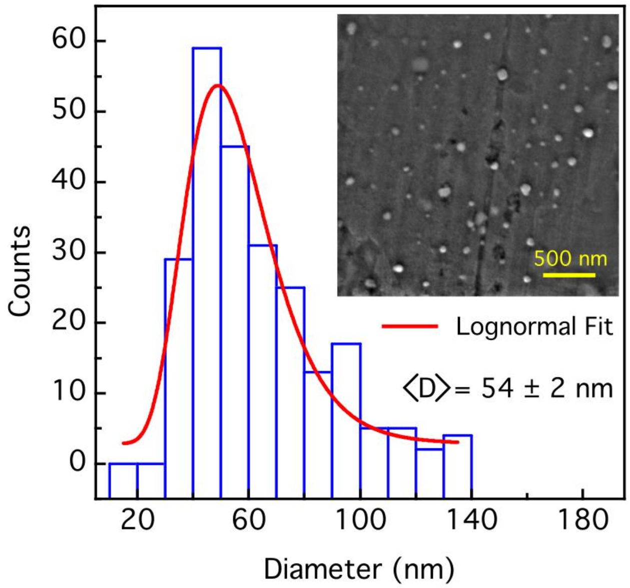

Figure S3. NPs size distribution obtained from FE-SEM images for NPs calcined at 630 ºC. The lognormal fit is also shown. *Inset:* FE-SEM image for the named sample.

Table S2. Raman mode shifts for each sample calcined at different temperatures. Reference Raman mode positions for single crystal bulk BFO have been included for comparisons.

	Our study (cm^{-1})					Bulk BFO [11] (cm^{-1})
Raman mode	400 ºC	460 ºC	500 ºC	580 ºC	630 ºC	
$E1$	71	71	70	71	72	77
$E2$	90	92	92	96	96	136
A_1-1	124	127	126	128	127	147
A_1-2	161	162	162	164	164	176
A_1-3	209	209	209	210	212	227
$E3$	256	258	258	261	261	265
$E4$	299	301	299	306	304	279
$E5$	347	347	347	348	347	351
$E6$	---	---	---	---	---	375
A_1-4	409	412	415	419	423	490
$E7$	467	467	466	466	467	437
$E8$	519	522	519	519	520	473
$E9$	628	629	621	622	---	525

Table S3. Best fitting results obtained from the M vs H curves (taken at 300 K and 5 K) for each sample calcined at the temperature indicated. Fittings were performed using modified Langevin function and assuming a lognormal-like distribution of magnetic moments[26]. N corresponds to the number of particles per unit mass and $\langle m \rangle$ to the average magnetic moment per particle. We also include a c parameter which is linear with the magnetic field and can be associated with the paramagnetic contribution to the NPs magnetization.

	300 K			5 K		
T_{cal} (^oC)	$N\left(\frac{1}{kg}\right)$	$\langle m \rangle$ (μ_B)	$c\left(\frac{emu}{g \cdot Oe}\right)$	$N\left(\frac{1}{kg}\right)$	$\langle m \rangle$ (μ_B)	$c\left(\frac{emu}{g \cdot Oe}\right)$
400	4.8×10^{18}	48	2.6×10^{-5}	4.8×10^{18}	50	1.6×10^{-5}
460	4.3×10^{18}	54	3.4×10^{-5}	4.3×10^{18}	58	2.6×10^{-5}
500	9.2×10^{17}	56	9.6×10^{-6}	9.2×10^{17}	59	1.5×10^{-5}
580	1.8×10^{17}	64	5.8×10^{-6}	1.8×10^{17}	68	1.5×10^{-5}
630	1.2×10^{17}	61	6.8×10^{-6}	1.2×10^{17}	70	3.2×10^{-5}
